# Berry Powder-Enriched Gluten-Free Extruded Snacks: Nutritional Quality and Antioxidant Potential

**DOI:** 10.3390/molecules31122074

**Published:** 2026-06-12

**Authors:** Anar Kurmanbayeva, Nazym Alzhaxina, Askhat Dalabayev, Nardias Balykbayev, Argyn Kaiyrkeldi

**Affiliations:** Kazakh Research Institute of Food and Processing Industry, Astana 010000, Kazakhstan; anara121579@gmail.com (A.K.); dalabaev_askhat@mail.ru (A.D.); popugaevpapug@gmail.com (A.K.)

**Keywords:** celiac disease, gluten-free products, extrusion, fruit-berry powder complex, antioxidant activity

## Abstract

The increasing prevalence of celiac disease underscores the need to develop nutritionally balanced, gluten-free snacks based on local raw materials. This study aimed to develop extruded gluten-free snacks based on corn, rice, buckwheat, and chickpea flours, enriched with a 5% blend of berry powders (sea buckthorn, blackcurrant, cranberry), and to evaluate their physicochemical, nutritional, and antioxidant properties. Snacks were produced via high-temperature short-time extrusion (120–160 °C). The results demonstrated that chickpea/corn formulations exhibited the highest initial protein content (13.87%), which remained robust after berry addition (9.14%), outperforming the starchy corn/rice control (7.61%). Enrichment significantly enhanced the functional profile: water-soluble antioxidants increased from 0.039 to 0.60–0.71 mg/g, and DPPH radical scavenging activity reached up to 61.8 ± 2.4%. Furthermore, the enriched snacks exhibited high retention of thermolabile compounds, including Vitamin C (up to 18.91 mg/100 g). Sensory evaluation confirmed excellent organoleptic acceptability without compromising texture. These findings quantitatively demonstrate that combining legume flours with berry powders enables the production of gluten-free extruded snacks with improved protein density, superior antioxidant potential, and moderate energy value (322–330 kcal/100 g), offering a functional alternative for specific dietary needs.

## 1. Introduction

Over the past 15–20 years, the global landscape has shifted such that many previously rare diseases, including autoimmune disorders, have reached epidemic proportions [1,2]. For many affected individuals, quality of life heavily depends on dietary management. In celiac disease, the only effective treatment currently available is a strict, lifelong gluten-free diet. However, this diet has significant drawbacks in practice. Complete avoidance of common cereals often leads to deficiencies in essential nutrients such as dietary fiber, protein, iron, and B-group vitamins [3,4]. Many commercially available gluten-free products fail to address these issues, as they are frequently high in refined starches, which can cause sharp spikes in blood glucose levels and promote inflammation [5,6].

One of the key approaches to improving the nutritional quality of gluten-free products is the rational selection of flour bases with complementary technological and nutritional properties. Rice flour is widely used in gluten-free formulations due to its neutral taste, hypoallergenic nature, and good digestibility; however, it is relatively poor in protein and micronutrients. Corn flour provides improved texture and contributes to the formation of a stable extrudate structure due to its starch composition. Chickpea flour (*Cicer arietinum*) is increasingly used as a functional ingredient because of its high protein content, significant levels of dietary fiber, and mineral elements such as iron, calcium, and magnesium. The combination of cereal and legume flours therefore represents a promising strategy for enhancing the biological value of gluten-free foods.

This issue is highly relevant in the Republic of Kazakhstan, as it is worldwide. For many years, the Central Asian region was considered low-risk for celiac disease. However, recent genetic screening studies have disproven this assumption, revealing that the frequency of HLA-DQ2/DQ8 allele carriage among the local population is comparable to that in European populations [7,8]. According to official statistical data from the Ministry of Healthcare of the Republic of Kazakhstan [9], the local dispensary registry for celiac disease grew nearly eightfold over ten years: from 37 patients in 2013 to 312 by the end of 2022.

Coupled with the high cost of imported products, this demographic shift underscores the necessity of developing domestic fortified foods using locally sourced gluten-free raw materials and berries [10].

High-temperature extrusion is a highly suitable technological approach for addressing challenges in specialized nutrition. Under thermomechanical processing at 130–180 °C, a complex of interrelated physicochemical transformations occurs, resulting in a porous structure with reduced moisture content and enhanced microbiological stability [11,12]. A promising direction involves the development of composite formulations incorporating grain-legume components. Specifically, chickpea flour (*Cicer arietinum*), which contains up to 24% protein and possesses significant mineral potential, is regarded as a functional ingredient capable of increasing the biological value of extruded products. Substituting a rice-corn base with chickpea flour raises in vitro protein digestibility while simultaneously reducing antinutrient concentrations [13,14]. Furthermore, legume proteins are hypothesized to form a more stable spatial matrix, potentially enhancing product shape retention upon exiting the extruder die, a protective mechanism similarly proposed in recent extrusion studies [11,13].

An equally important aspect is the fortification of snacks with antioxidant ingredients. Sea buckthorn (*Hippophae rhamnoides*), blackcurrant (*Ribes nigrum*), and cranberry (*Vaccinium macrocarpon*) are rich sources of biologically active compounds, including polyphenols, flavonoids, vitamin C, and organic acids [12,15,16]. The incorporation of these berry powders into extruded snacks represents a promising strategy for increasing both antioxidant capacity and overall nutritional value [16]. Despite the common belief that ascorbic acid is completely degraded during high-temperature processing, contemporary data indicate partial preservation of polyphenols and vitamins due to the protective effect of dietary fiber from plant raw materials [13,17]. Furthermore, berry component dosages in the range of 5–15% do not compromise the textural characteristics of the extrudate [15,16].

Furthermore, buckwheat (*Fagopyrum esculentum*) is a highly valuable pseudocereal for gluten-free diets. It is distinguished by a well-balanced amino acid profile, a high content of dietary fiber, and a unique set of bioactive compounds, particularly the antioxidant rutin. The incorporation of buckwheat into snack formulations not only improves the structural and mechanical properties of the extrudates but also significantly increases their functional value and antioxidant capacity [17].

To address these challenges, the aim of this study was to develop gluten-free extruded snacks based on domestic corn, rice, buckwheat, and chickpea flour fortified with a composite berry powder. Furthermore, we evaluated the impact of this fortification on the proximate composition, vitamin profile (B-group and Vitamin C), and antioxidant potential of the final products to ensure their suitability for the specialized nutrition of children with celiac disease.

## 2. Results

### 2.1. Physicochemical Properties and Energy Value of Extruded Snacks

To assess the physicochemical properties of the extruded gluten-free snacks, samples without and with berry powder addition were analyzed, enabling observation of changes in the mass fractions of protein, carbohydrates, fat, ash, and energy value (Table 1).

Analysis of the physicochemical parameters (Table 1) revealed distinct differences among formulations based on various flour compositions.

The control sample (rice/corn) was characterized by the lowest protein mass fraction (7.61 ± 0.06%), the highest carbohydrate content (70.09 ± 0.55%), the lowest ash content (0.83 ± 0.004%), and an energy value of 337.08 kcal/100 g.

The highest protein content was recorded in the chickpea/corn mixture (13.87 ± 0.11%), with ash content of 2.05 ± 0.06% and energy value of 359.62 kcal/100 g. Buckwheat-based samples occupied an intermediate position (10.25 ± 0.09% protein).

The addition of 5% berry composition led to a reduction in macronutrient mass fractions due to structural displacement. In the chickpea/corn/berries sample, protein content decreased to 9.14 ± 0.10%, and fat to 4.26 ± 0.02%.

The energy value of the enriched samples ranged from 330.89 kcal/100 g (chickpea/corn/berries), 322.66 kcal/100 g (buckwheat/corn/berries), to 330.83 kcal/100 g (rice/corn/berries).

Thus, the incorporation of berry powders resulted in a slight reduction in the product’s energy density.

### 2.2. Vitamin Composition of Extruded Snacks

To comprehensively evaluate the biological value of the developed extruded snacks, their vitamin profile was analyzed, including B-group vitamins (B1, B2, B3, B5, B6, Bc/folate) and vitamin C. These micronutrients play a key role in energy metabolism, nervous system function, and antioxidant defense, which is particularly important for formulating diets for children with celiac disease on a long-term gluten-free regimen.

Vitamin content was determined post high-temperature short-time extrusion to assess retention under thermomechanical processing and the contribution of the composite berry powder mixture (black currant, cranberry, sea buckthorn) to fortification of the final product. The obtained results are presented in Table 2 and reflect the influence of the flour base type and berry component on the micronutrient profile of the extrudates. The moisture content of the extrusion mixture plays a critical role in preserving thermolabile compounds, particularly vitamin C. Higher moisture levels can act as a plasticizer in the extruder barrel, reducing mechanical shear and melt temperature, thereby mitigating the severe thermal degradation of sensitive vitamins.

Vitamin Analysis (Table 2) revealed that the micronutrient profile depended on the type of flour base and the presence of berry additives. Berry powder was characterized by high contents of: vitamin C −560.08 ± 6.7 mg/100 g, niacin −2.25 ± 0.405 mg/100 g, and pantothenic acid −1.97 ± 0.394 mg/100 g.

In extruded samples, vitamin C content ranged from 10.47 to 18.91 mg/100 g, and folic acid content ranged from 0.011 to 0.017 mg/100 g. The highest values of thiamine (0.444 ± 0.08 mg/100 g) and niacin (2.18 ± 0.39 mg/100 g) were recorded in the corn/buckwheat/berries sample.

Among the investigated vitamins, niacin was detected in the highest concentrations (1.35–2.18 mg/100 g) compared to other B-group vitamins. This indicates its robust presence in the final extrudate under high-temperature short-time extrusion conditions.

### 2.3. Antioxidant Activity

The aim of this study was to investigate the influence of a complex of fruit and berry powders derived from blackcurrant, cranberry, and sea buckthorn on the antioxidant activity of gluten-free berry snacks (Table 3).

Data from Table 3 demonstrated a significant increase in antioxidant capacity across all assays upon the addition of the berry composition. Water-soluble antioxidants increased from 0.020 to 0.039 mg/g to 0.60–0.71 mg/g, while fat-soluble antioxidants rose from 0.002 to 0.003 mg/g to 0.06–0.09 mg/g. This enhancement was strongly supported by the DPPH and FRAP assays; radical scavenging activity (DPPH) surged from 18.4 to 24.7% in control samples to 55.6–61.8% in enriched snacks, and reducing power (FRAP) increased more than threefold (up to 1.84 mmol Fe^2+^/100 g in the Corn/Rice/Berry sample).

To further characterize the specific bioactive profile responsible for this enhanced antioxidant potential, HPLC profiling of phenolic compounds was performed (Table 4). The results show that the incorporation of berry powders provided high concentrations of major identified phenolic compounds, including anthocyanins, quercetin, and chlorogenic acid, which directly correspond to the elevated radical scavenging activity and reducing power observed in the extrudates.

### 2.4. Sensory Properties

The sensory panel established that all three formulations (Rice-, Buckwheat-, and Chickpea-based snacks) exhibited similar high levels of acceptability, with the characteristic traits of the berry additives being successfully preserved in each matrix (Table 5).

## 3. Discussion

### 3.1. Analysis of Physicochemical Properties of Extruded Gluten-Free Snacks

The control sample based on a rice and corn flour blend exhibited the lowest protein content (7.61 ± 0.06%) alongside the highest carbohydrate content (70.09 ± 0.55%) and the lowest ash content (0.83 ± 0.004%). From a physicochemical perspective, the predominance of starch fractions (amylose and amylopectin) in the rice-corn matrix promoted a high degree of gelatinization during thermomechanical processing (extrusion); however, this resulted in a product with “empty” caloric density.

To overcome this technological limitation, modern research proposes enriching starch-based formulations with legume-derived proteins using extrusion cooking. In particular, the incorporation of garbanzo (chickpea) flour into corn-based systems has been shown to improve the nutritional profile of gluten-free extruded snacks, including increased protein content and enhanced functional properties, depending on formulation composition and processing conditions. Furthermore, the addition of chickpea flour to rice-based matrices under mild extrusion conditions significantly enhances the protein profile and antioxidant activity while maintaining the structural stability of the extrudate. In the present study, the incorporation of chickpea flour as a protein enricher (chickpea/corn blend) resulted in a protein content of 13.87 ± 0.11% and ash content of 2.05 ± 0.06% [18,19].

The high protein level in chickpeas facilitated the formation of additional intermolecular bonds (disulfide and hydrogen bonds) with corn starch during extrusion, thereby strengthening the pore walls of the final snack. These values substantially outperformed traditional analogs and demonstrated that legume incorporation represents a highly effective strategy for nutritional enhancement, eliminating the need for costly protein isolates. Samples based on buckwheat flour occupied an intermediate position (10.25 ± 0.09% protein), consistent with current data on the high physicochemical potential of pseudocereals, which are rich in complete amino acid profiles and minerals [20].

The addition of berry powders to gluten-free snacks is a well-established approach for functionalizing food systems and enhancing their antioxidant potential; however, this inevitably modifies the base macrostructure of the extruded product [21]. In comparable studies, the effects of various dried berries (elderberry, blackberry, apple) on corn snacks were investigated; additions exceeding 10% were found to significantly dilute the starch-protein matrix and reduce radial expansion of the extrudate [22]. This is attributed to the high water-binding capacity of dietary fibers and pectins in berries, which compete with starch for free moisture in the extruder barrel, thereby hindering complete gelatinization. Similar physicochemical shifts were reported in extrudates incorporating blueberry pomace powder: increasing powder levels led to altered chemical properties, increased hardness, and reduced macronutrient density [23].

Analysis of the data presented in Table 1 indicated that the addition of 5% composite fruit and berry powder mixture (sea buckthorn, blackcurrant, cranberry) led to a predictable reduction in the proportions of major macronutrients due to a structural dilution effect. Thus, in the “chickpea flour/corn flour/berry powder mixture” sample, protein content decreased from 13.87 ± 0.11% to 9.14 ± 0.10%, and fat content decreased from 5.30 ± 0.04% to 4.26 ± 0.02%. Similar reductions in carbohydrate and protein contents upon addition of fruit powders (e.g., chokeberry, açaí) have been observed in comparable scientific studies [24]. While a direct 5% substitution mathematically accounts for only a partial reduction, the more pronounced decrease in the relative protein mass fraction is attributed to complex mass-balance shifts during extrusion. The high water-binding capacity of berry fibers and sugars alters the moisture flash-off dynamics at the die, leading to shifts in final residual moisture and a relative proportional redistribution of all proximate macronutrients within the dry weight basis.

Nevertheless, despite the slight structural dilution caused by the berry powder addition, the final protein levels in the enriched chickpea-corn-berry (9.14 ± 0.10%) and buckwheat-corn-berry (8.51 ± 0.07%) snacks remained consistently higher than in the baseline starchy control (7.61 ± 0.06%). This anticipated minor reduction in base macronutrients represents a technologically justified compromise; the incorporation of sea buckthorn, blackcurrant, and cranberry successfully provided thermostable polyphenols, organic acids, and essential vitamins, shifting the product from a simple carbohydrate snack to a valuable fortified food.

In the context of the global increase in celiac disease and metabolic disorders, the energy value of gluten-free products is a critical parameter for consumers [17]. Frequent use of lipids in commercial snacks to enhance sensory qualities often results in excessive caloric density. As shown in Table 1, the baseline blends exhibited energy values ranging from 337.08 kcal/100 g (rice/corn) to 359.62 kcal/100 g (chickpea/corn). The higher value for the chickpea blend was attributable to the naturally elevated lipid content in legumes (5.30 ± 0.04%), which consisted predominantly of valuable unsaturated fatty acids, in contrast to the added trans-isomers or saturated fats commonly used in mass-market products.

Furthermore, the incorporation of the berry powder mixture allowed for the maintenance of a moderate energy density. The energy value across all three enriched blends stabilized within a narrow range of 322.66–330.89 kcal/100 g. The reductions in fat and carbohydrate proportions in the enriched samples compensated for the initial differences among the base flour matrices. This confirms that the proposed formulation and extrusion parameters enable the production of fortified gluten-free snacks with standardized, tightly controlled, and moderate caloric content, fully aligning with contemporary nutritional requirements.

### 3.2. Analysis of Vitamin Composition in Extruded Vitamin-Enriched Snacks

Comparative analysis of the vitamin composition demonstrated a pronounced influence of both formulation and extrusion processing on micronutrient retention. The berry powder (blackcurrant, cranberry, sea buckthorn) was characterized by exceptionally high vitamin C content (560.08 ± 6.7 mg/100 g), along with elevated concentrations of B-group vitamins, particularly niacin (2.25 ± 0.405 mg/100 g) and pantothenic acid (1.97 ± 0.394 mg/100 g). However, its incorporation into extruded matrices was accompanied by partial degradation of thermolabile compounds.

Among the extruded samples, the highest thiamine (0.444 ± 0.08 mg/100 g) and niacin (2.18 ± 0.39 mg/100 g) contents were observed in the corn/buckwheat/berry powder formulation. These findings align with studies on enriched gluten-free snacks [25,26], which highlight the high functional value of pseudocereals as stable sources of B-group vitamins during thermal processing. In the corn/chickpea/berry powder sample, lower values were recorded for pantothenic acid (0.489 ± 0.097 mg/100 g) and pyridoxine (0.451 ± 0.090 mg/100 g). This suggested greater sensitivity of these vitamins to thermomechanical degradation in protein-rich systems. As demonstrated in recent research [24,27], elevated protein content intensifies Maillard reactions during extrusion, which can lead to binding and co-degradation of certain micronutrients.

The theoretical Vitamin C content in the raw mixtures prior to extrusion was calculated to be approximately 28.00 mg/100 g (based on a 5% inclusion rate of berry powder containing 560.08 mg/100 g of ascorbic acid). Following HTST extrusion, the Vitamin C content in the fortified snacks ranged from 10.47 to 18.91 mg/100 g. This demonstrates a significant vitamin retention rate ranging from 37.4% to 67.5%, depending on the formulation. The highest stability of Vitamin C was observed in the chickpea-based matrix (18.91 mg/100 g), suggesting a protective effect of the legume protein-starch complex during thermal processing. While ascorbic acid is highly sensitive to elevated temperatures and shear stresses [28,29], the encapsulation of thermolabile compounds within the extruded matrix allowed for substantial retention. A similar trend was noted for folic acid (vitamin B9), with residual concentrations of only 0.011–0.017 mg/100 g, confirming its instability under harsh thermo-oxidative conditions [30].

It is important to note that absolute retention rates cannot be definitively calculated in this study due to the absence of pre-extrusion vitamin quantification in the raw blends. However, the remarkably high final concentrations of Vitamin C and B-group vitamins in the extrudates strongly suggest their effective preservation. The observed stability during the high-temperature short-time (HTST) process aligns with recent literature, which demonstrates that short residence times, combined with the plasticizing effect of optimal moisture content, can significantly mitigate the thermal degradation of heat-sensitive bioactive compounds [30,31]. Furthermore, the robust presence of niacin (1.35–2.18 mg/100 g) aligns with literature documenting its high thermal resistance, potentially aided by release from bound forms during starch gelatinization [31,32].

Thus, extrusion represents a critical factor determining vitamin retention levels. Despite the short residence time in the extruder, the combination of high temperature and mechanical shear substantially affects thermolabile compounds [33]. The addition of berry powder partially compensates for micronutrient losses, underscoring the promise of developing fortified gluten-free extruded snacks. Optimization of extrusion parameters (temperature profile, feedstock moisture, screw speed) appears essential for improving vitamin retention and enhancing the product’s functional value.

### 3.3. Antioxidant Activity of Gluten-Free Extruded Snacks

Numerous studies have demonstrated that fortifying products with bioactive additives from antioxidant-rich fruits and vegetables, which also provide vitamins and dietary fiber, is an effective strategy for improving nutritional value [25,34,35]. Fruits and vegetables exert protective effects against various diseases, including cancer and cardiovascular pathologies, owing to the activity of their bioactive components [36,37].

Previous research has examined extruded snacks enriched with fruit by-products; for instance, the addition of 10% raspberry pomace to gluten-free matrices enhanced antioxidant activity twofold, while cherry and chokeberry extracts in corn snacks increased DPPH inhibition to approximately 40–45% [38,39]. In direct quantitative comparison, our study achieved a remarkably higher DPPH inhibition (up to 61.8 ± 2.4%) using only a 5% inclusion rate of the composite berry powder, quantitatively highlighting the superior functional efficacy of the selected sea buckthorn, blackcurrant, and cranberry blend. The effects of extrusion and local fruit/berry bioactive additives on the antioxidant activity of gluten-free snacks have also been investigated [40,41,42], including novel 3D technologies that revealed synergistic and antagonistic interactions among bioactive components [43].

Analysis of the data in Table 3 indicated a pronounced dependence between the vitamin composition of extruded gluten-free snacks and their water- and fat-soluble antioxidant activity levels. Incorporation of berry powders into the formulation resulted in a multifold increase in antioxidant potential compared to control samples without additives.

For water-soluble antioxidants, levels in snacks without berry powder addition ranged from 0.020 to 0.039 mg/g, whereas in samples with berry powder, this increased to 0.60–0.71 mg/g. The maximum water-soluble antioxidant activity was recorded in corn- and rice-based snacks (0.71 mg/g), correlating with relatively high contents of vitamins B_6_ and C (0.734 and 10.47 mg/100 g, respectively; Table 1). Vitamin C is recognized as a key water-soluble antioxidant, explaining the observed enhancement. The particularly high level of water-soluble antioxidants in the corn/rice matrix suggests that this specific carbohydrate-dense structure provides a more protective environment against thermal and oxidative degradation during extrusion compared to protein-rich matrices.

Berry powders exhibited exceptionally high water-soluble antioxidant levels (1.92 mg/g), consistent with their elevated vitamin C content (560.08 mg/100 g) as well as folic acid and other bioactive compounds. This confirms their dominant contribution to the antioxidant properties of the final extruded products, despite partial degradation of thermolabile compounds during extrusion.

Fat-soluble antioxidant activity in the investigated samples also increased significantly upon berry powder addition. In control snacks without additives, this parameter did not exceed 0.002–0.003 mg/g, whereas in enriched samples it reached 0.06–0.09 mg/g. The highest value was noted in the “corn/buckwheat” snacks, primarily associated with the presence of polyphenolic compounds from the berry powder, as well as fat-soluble antioxidants such as tocopherols and carotenoids derived from both plant raw materials and berry components.

Although berry powders contained high concentrations of fat-soluble antioxidants (0.38 mg/g), their levels in the final snacks were lower, likely due to redistribution and partial degradation of lipophilic compounds under high-temperature extrusion conditions. Nevertheless, even after processing, a statistically significant increase in fat-soluble antioxidant activity was observed relative to controls.

Overall, the data demonstrate that the elevated antioxidant activity in extruded gluten-free snacks arises from the combined effects of vitamin C, B-group vitamins, and bioactive compounds from berry powder. These results support the viability of using berry powders as a functional ingredient to develop products with enhanced antioxidant and biological value.

### 3.4. Sensory Characteristics Interpretation

The pairwise comparison of control and fortified formulations reveals that the organoleptic impact of berry powder is highly dependent on the baseline properties of the raw flour matrix. In the rice-based system, which inherently lacks intense flavor and color, the 5% berry powder acted as a prominent quality driver (*p* < 0.05), substantially lifting the visual and flavor attributes. Conversely, the buckwheat- and chickpea-based controls already possessed strong native earthy notes, distinct aromas, and darker pigmentations. In the buckwheat matrix, the introduction of the berry additive slightly competed with the robust characteristic notes of the grain, explaining the minor decrease in color and aroma scores. However, the taste attributes for all fortified samples consistently improved to 4.40 (*p* < 0.01), demonstrating that the sweet-sour polyphenolic profile of the berries efficiently masked the flat, bitter, or potentially beany aftertastes of raw pulses and pseudo-cereals.

The lack of statistical significance in texture variations (*p* = 0.315) across all six formulations carries profound technological importance for the functional food industry. It demonstrates that the addition of a 5% fiber-rich berry fraction did not disrupt starch gelatinization or nucleated cell expansion dynamics during the extrusion process. Typically, the inclusion of fruit or berry pomace restricts water binding, leading to dense and unappealing extrudates. In this study, the chosen precision processing parameters successfully balanced the preservation of heat-sensitive volatile pigments and natural organic acids with the retention of excellent structural crispiness, ensuring a highly viable consumer profile.

### 3.5. Limitations of the Study and Future Perspectives

While the present study demonstrates the high potential of berry-enriched gluten-free extruded snacks, several limitations should be acknowledged. First, the evaluation of vitamin retention was conducted without measuring the exact pre-extrusion vitamin concentrations in the raw blends; therefore, absolute retention rates could not be definitively calculated, and the discussed thermal stability is primarily based on comparative final values and existing literature. Second, the textural evaluation relied exclusively on a trained sensory panel. Future studies should incorporate instrumental texture profile analysis (TPA) to provide quantitative mechanical parameters (e.g., hardness, crispiness index). Additionally, this study focused on the immediate post-extrusion characteristics and did not include long-term storage stability tests or in vitro/in vivo bioavailability assessments of the incorporated polyphenols. Finally, while the 5% berry inclusion demonstrated optimal sensory results, further optimization of the extrusion parameters using response surface methodology (RSM) could be beneficial to maximize the retention of specific bioactive compounds.

## 4. Materials and Methods

### 4.1. Raw Materials and Sample Preparation

Extruded snacks were produced using domestically bred grain and legume processing products (Republic of Kazakhstan). The basic gluten-free flour blends (corn/rice, corn/buckwheat, and corn/chickpea) were prepared using a mass ratio of 75:25. This ratio of cereal to legume/pseudocereal flour was selected based on preliminary laboratory trials to ensure adequate starch gelatinization and optimal radial expansion, while maximizing protein enrichment. The gluten-free blends consisted of corn flour variety “Kazakhstansky 43 TV”, round-grain rice flour variety “Marzhan”, buckwheat flour variety “Shortandinskaya krupnozernaya”, and chickpea flour variety “Miras 07”. As a functional component, a composite mixture of fruit and berry powders was used, derived from sea buckthorn (*Hippophae rhamnoides*), blackcurrant (*Ribes nigrum*), and cranberry (*Vaccinium macrocarpon*), all cultivated in Kazakhstan. The 5% inclusion level for the berry powder mixture was determined to be optimal; preliminary tests indicated that higher concentrations (>10%) significantly disrupted the extrudate matrix and increased hardness due to the excessive water-binding capacity of the berry dietary fibers and high sugar content. Berry raw materials underwent convective drying at 55 and 70 °C ((KT-1908, Kitfort, St. Petersburg, Russia) for 5–20 h until constant mass was achieved, followed by grinding to a particle size ≤0.5 mm (using a Hawos Pegasus Laboratory Mill, Hawos Kornmühlen GmbH, Bad Homburg, Germany). All raw materials were preliminarily inspected for quality and moisture content prior to processing.

### 4.2. Chemicals and Reagents

All chemical reagents and standards were of analytical grade. High-purity standards of B-group vitamins and L-ascorbic acid (purity ≥ 98%) were purchased from Sigma-Aldrich (St. Louis, MO, USA). HPLC-grade methanol, acetonitrile, and formic acid were obtained from Merck (Darmstadt, Germany). Ultrapure water (18.2 MΩ·cm) was produced using a Milli-Q system (Millipore, Bedford, MA, USA). All solutions were filtered through 0.22 μm membrane filters prior to chromatographic analysis.

### 4.3. Extrusion Processing

Experimental samples were produced by high-temperature short-time (HTST) extrusion using a single-screw laboratory extruder (Model TSE-30, Jinan Saibainuo Machinery, Jinan, China). Prior to extrusion, multicomponent blends were moistened to 15–18% by adding the calculated amount of water and equilibrated to ensure uniform moisture distribution.

The process was conducted at a screw speed of 200 rpm and a feed rate of 25 kg/h. The extruder barrel featured three heating zones with a temperature profile of 120–160 °C. The die diameter was 3.0 mm. Technological parameters (temperature profile and feed rate) were adjusted to achieve stable expansion while minimizing thermal degradation of bioactive compounds. The resulting extrudates were cooled at room temperature for 15–20 min. To ensure the reproducibility of the results, the extrusion process was conducted in three independent production batches on different days (biological replicates). The processing order of the formulations was randomized. From each batch, samples were collected in triplicate for analytical measurements (analytical replicates).

### 4.4. Proximate Composition Analysis

All determinations were performed in triplicate, and results were expressed as mean ± standard deviation (SD), with statistical significance evaluated as described above (*p* < 0.05). The basic nutritional profile of the extruded snacks was determined in accordance with the official standard methods of the International Organization for Standardization (ISO). Protein content was determined by the Kjeldahl method (ISO 20483) using a conversion factor of 6.25 [44]. Fat content was measured by Soxhlet extraction with hexane as the solvent (ISO 11085) [45]. Ash content was determined by dry ashing at 500–600 °C until constant mass was achieved (ISO 2171) [46]. Carbohydrate content was calculated by difference. All analytical results for proximate composition were calculated and expressed on a dry weight basis (% DW) to ensure comparability between samples. Energy value was calculated using the formula:

(1) E = 4P + 9F + 4C

where *E* is energy value (kcal/100 g), *P* is protein (%), *F* is fat (%), and *C* is carbohydrates (%).

### 4.5. Analysis of Vitamin Composition

B-group vitamins (B1, B2, B3, B5, B6, and Bc/folates) were determined in accordance with standardized analytical procedures. Specifically, thiamine (B1), riboflavin (B2), niacin (B3), and pyridoxine (B6) were quantified following EN 14122 [47], EN 14152 [48], EN 15652 [49], and EN 14164 [50], respectively. Pantothenic acid (B5) was determined according to ISO 20639:2015 [51]. Folates (vitamin Bc) were analyzed using a microbiological assay in accordance with EN 14131 [52].

For the determination of thiamine, riboflavin, niacin, pantothenic acid, and pyridoxine, chromatographic separation was performed using a high-performance liquid chromatograph (Shimadzu LC-20 Prominence system, Kyoto, Japan) equipped with a reversed-phase C18 column (250 × 4.6 mm, 5 μm particle size) and a UV detector. The column temperature was maintained at 30 °C. Separation was achieved using a linear gradient elution with a mobile phase consisting of 0.1% formic acid in water (A) and methanol (B). The gradient program was as follows: 0–2 min, 5% B; 2–15 min, 5–60% B; 15–20 min, 60–100% B; followed by 5 min of re-equilibration. The flow rate was set at 1.0 mL/min and detection was carried out at wavelengths specific for each vitamin (254–280 nm). Calibration curves were constructed using external standard solutions over at least five concentration levels (R^2^ ≥ 0.999), ensuring linearity and accuracy of quantification.

Vitamin C (ascorbic acid) content was determined using a titrimetric method based on redox reaction with 2,6-dichlorophenolindophenol (DCPIP) [53].

Approximately 5.0 g of the homogenized sample was extracted with 50 mL of 3% metaphosphoric acid solution to stabilize ascorbic acid and prevent oxidation. The mixture was homogenized for 2–3 min and filtered through Whatman No. 1 filter paper.

An aliquot (10 mL) of the filtrate was titrated with standardized DCPIP solution until a persistent light pink color appeared and remained for at least 15 s. A reagent blank was performed under identical conditions.

The vitamin C content was calculated according to the following equation:

(2) $$\mathrm{Vitamin}\;C\;(\mathrm{mg}/100\;g)\;=\frac{V\times F\times 100}{m}$$

where *V* is the volume of DCPIP solution used for titration (mL),

*F* is the factor corresponding to mg of ascorbic acid per 1 mL of DCPIP solution, *m* is the sample mass (g).

All determinations were performed in triplicate, and results were expressed as mean ± standard deviation.

### 4.6. Antioxidant Activity Determination

For the antioxidant activity assay, sample extracts were prepared as follows: 1.0 g of finely ground snack was extracted with 50 mL of bidistilled water under continuous stirring for 30 min at room temperature. The mixture was then centrifuged at 4000 rpm for 15 min, and the supernatant was filtered through a 0.22 μm membrane filter. Antioxidant activity was measured amperometrically using a Tsvet Yauza-01-AA instrument (NPO Khimavtomatika, Moscow, Russia) by recording the anodic current generated from electrochemical oxidation of antioxidant compounds at a fixed potential [54]. The amperometric method allows rapid determination of the total antioxidant capacity of complex food matrices by measuring the electrochemical response of oxidizable antioxidant compounds present in the sample extract. Results were expressed in equivalents of a standard antioxidant based on the calibration curve.

For the determination of fat-soluble antioxidants, a similar extraction principle was applied using hexane as the solvent. Briefly, 1 g of the crushed snack sample was mixed with 10 mL of hexane, vortexed thoroughly, and centrifuged at 4000 rpm for 10 min. The resulting supernatant was collected and used for the amperometric measurement.

Gallic acid was used as a reference standard for calibration of antioxidant activity, and results were expressed as mg gallic acid equivalents (GAE) per g of sample based on the corresponding calibration curve.

To strengthen the evaluation of antioxidant potential, additional antioxidant assays including DPPH radical scavenging activity and FRAP reducing power assay were performed according to internationally accepted analytical protocols [55,56]. Briefly, the DPPH assay was conducted by mixing the sample extract with a methanolic DPPH solution, and absorbance was measured at 515 nm. The FRAP assay was based on the reduction in the Fe^3+^-TPTZ complex to the blue-colored Fe^2+^-TPTZ, with absorbance recorded at 593 nm. Furthermore, the phenolic profile of berry-enriched extruded snacks was analyzed by high-performance liquid chromatography (HPLC) according to ISO 14502-1 [57] and ISO 14502-2 [58] methodologies. The chromatographic analysis enabled the identification and quantification of major phenolic compounds, including phenolic acids, flavonoids, and anthocyanins responsible for the antioxidant activity of the developed products. Identification of individual phenolic compounds was performed by directly comparing their chromatographic retention times and UV spectra with those of highly pure authentic commercial standards.

### 4.7. Sensory Evaluation

Sensory analysis of the developed gluten-free snacks (Rice/Corn/Berry, Buckwheat/Corn/Berry, and Chickpea/Corn/Berry) was conducted in strict accordance with the international standard ISO 6658:2017 (Sensory analysis—Methodology—General guidance) [59]. The evaluation was performed at the dedicated sensory laboratory of the Kazakh Research Institute of Food and Processing Industry.

Prior to participation, all panelists provided informed consent. The sensory evaluation involved only standard, safe food ingredients and was conducted in accordance with the institutional ethical guidelines.

The trained panel consisted of 10 expert assessors (5 male, 5 female, aged 25–45). All selected panelists are professional researchers and specialists in the food industry, holding relevant academic degrees in food science and processing technology. They were selected, trained, and formally qualified according to the guidelines of ISO 8586:2023 [60]. Prior to the main evaluation, a training session was conducted to familiarize the expert panel with the specific descriptive attributes of berry-enriched extrusion matrices.

The descriptive profiling method was used to assess five core quality attributes: appearance (shape and surface), color, texture (crunchiness), taste, and aroma. Each attribute was evaluated for its intensity and typicality using a 5-point hedonic scale (1 = poor/unacceptable, 5 = excellent). The total sensory score was calculated as the sum of these five individual attributes, resulting in a maximum possible score of 25 points to ensure comprehensive statistical differentiation.

### 4.8. Statistical Analysis

All experiments and analytical measurements were performed in at least three independent replicates (n ≥ 3). The results are presented as the mean value ± standard deviation (SD). Statistical data processing was performed using Statistica 12.0 software (StatSoft Inc., Tulsa, OK, USA). Prior to analysis, the normality of data distribution and the homogeneity of variances were verified using the Shapiro–Wilk and Levene’s tests, respectively. To determine the statistical significance of differences between the mean values of the independent groups, a one-way analysis of variance (ANOVA) was applied. When significant main effects were identified, Tukey’s honestly significant difference (HSD) post hoc test was utilized for multiple comparisons.

## 5. Conclusions

As a result of this study, gluten-free extruded snacks were developed based on domestically sourced grain and legume raw materials (Republic of Kazakhstan) enriched with 5% composite berry powder mixture (*Hippophae rhamnoides, Ribes nigrum, Vaccinium macrocarpon*), followed by comprehensive evaluation of their physicochemical, vitamin, and antioxidant characteristics.

Incorporation of chickpea flour into the corn matrix provided the highest protein content (13.87%), substantially exceeding that of the rice-corn control (7.61%). Addition of the berry composition led to a predictable reduction in macronutrient proportions due to structural dilution; however, final protein values in the enriched samples (8.51–9.14%) remained higher than in the control. Energy value in the fortified samples stabilized within a narrow range of 322.66–330.89 kcal/100 g, indicating the formation of products with moderate and standardized caloric density.

The formulated snacks demonstrated a rich profile of B-group vitamins and ascorbic acid. Specifically, the use of a chickpea-based flour matrix resulted in the highest final concentration of vitamin C (18.91 mg/100 g) among the studied samples. While the exact thermal retention rates could not be calculated without pre-extrusion data, the final concentrations meet a significant portion of the daily nutritional requirements, confirming the nutritional viability of the chosen ingredients for specialized gluten-free diets.

The most pronounced enrichment effect was observed in antioxidant activity. Water-soluble antioxidant content increased from 0.020–0.039 to 0.60–0.71 mg/g, and fat-soluble antioxidant content from 0.002–0.003 to 0.06–0.09 mg/g. These results highlight the dominant contribution of polyphenolic compounds from the berry complex to the antioxidant potential of extrudates, even post-processing.

Thus, taking into account the comparison with control samples without berry powder, the developed formulation based on legume–grain matrices and local berry ingredients enables the production of gluten-free extruded snacks with enhanced protein value, increased antioxidant activity, and controlled energy density.

The obtained results should be interpreted considering the presence of control formulations, which allows for a more reliable assessment of the effect of berry powder addition on the nutritional and functional properties of the products.

This approach holds practical significance for expanding the range of fortified specialized nutrition products, including those for children with celiac disease, while promoting rational utilization of regional raw material resources.

## Figures and Tables

**Table 1:** Physicochemical parameters and energy value of the developed extruded snacks (expressed on a dry weight basis, % DW).

Parameter	Corn/Chickpea (Control)	Corn/Buckwheat (Control)	Corn/Rice (Control)	Mix of fruit and Berry Powders	Corn/Chickpea/Berry	Corn/Buckwheat/Berry	Corn/Rice/Berry	*p*-Value
Mass fraction of protein, %	13.87 ± 0.11 ^a^	10.25 ± 0.09 ^b^	7.61 ± 0.06 ^e^	3.20 ± 0.05 ^g^	9.14 ± 0.10 ^c^	8.51 ± 0.07 ^d^	6.69 ± 0.09 ^f^	<0.001
Mass fraction of carbohydrates, %	64.11 ± 0.44 ^f^	66.24 ± 0.68 ^d^	70.09 ± 0.55 ^b^	78.50 ± 0.60 ^a^	63.99 ± 0.76 ^g^	65.18 ± 0.74 ^e^	69.83 ± 0.59 ^c^	<0.001
Mass fraction of fat, %	5.30 ± 0.04 ^a^	3.77 ± 0.02 ^c^	2.92 ± 0.01 ^e^	1.80 ± 0.02 ^g^	4.26 ± 0.02 ^b^	3.10 ± 0.03 ^d^	2.75 ± 0.0 ^f^	<0.001
Mass fraction of ash, %	2.05 ± 0.06 ^b^	1,66 ± 0.07 ^d^	0.83 ± 0.004 ^g^	3.50 ± 0.04 ^a^	1.80 ± 0.02 ^c^	1.40 ± 0.05 ^e^	1.07 ± 0.04 ^f^	<0.001
Energy value, kcal	359.62 ^a^	339.89 ^b^	337.08 ^c^	314.86 ^g^	330.89 ^d^	322.66 ^f^	330.83 ^e^	<0.001

Values are presented as mean ± SD (*n* = 3). Energy value was calculated using the Atwater general factors (4 kcal/g for protein and carbohydrates, 9 kcal/g for fat). Different superscript letters (a–g) within the same row indicate statistically significant differences, and the corresponding *p*-values for each parameter are shown in the rightmost column (*p* < 0.05).

**Table 2:** Comparative analysis of the vitamin composition of extruded fortified snacks (mg/100 g).

Sample	Vitamin B1 (Thiamine) (mg/100 g)	Vitamin B2 (Riboflavin) (mg/100 g)	Vitamin B3 (Niacin) (mg/100 g)	Vitamin B5 (Pantothenic Acid) (mg/100 g)	Vitamin B6 (Pyridoxine) (mg/100 g)	Vitamin Bc (Folic Acid) (mg/100 g)	Vitamin C (Ascorbic Acid) (mg/100 g)
Corn/Rice/Berry	0.303 ± 0.06 ^b^	0.109 ± 0.04 ^c^	1.62 ± 0.29 ^b^	1.305 ± 0.261 ^b^	0.734 ± 0.146 ^ab^	0.015 ± 0.003 ^b^	10.47 ± 0.37 ^d^
Corn/Buckwheat/Berry	0.444 ± 0.08 ^a^	0.166 ± 0.069 ^b^	2.18 ± 0.39 ^a^	1 63 ± 0.232 ^a^	0.668 ± 0.133 ^b^	0.011 ± 0.002 ^b^	13.01 ± 0.51 ^c^
Corn/Chickpea/Berry	0.290 ± 0.058 ^b^	0.074 ± 0.031 ^c^	1.35 ± 0.243 ^b^	0.489 ± 0.097 ^c^	0.451 ± 0.090 ^c^	0.017 ± 0.0002 ^b^	18.91 ± 0.51 ^b^
Berry powder mixture	0.302 ± 0.06 ^b^	0.219 ± 0.091 ^a^	2.25 ± 0.405 ^a^	1.97 ± 0.394 ^a^	0.783 ± 0.156 ^a^	0.069 ± 0.014 ^a^	560.08 ± 6.7 ^a^
*p*-value	<0.05	<0.05	<0.05	<0.05	<0.05	<0.001	<0.001

Values are presented as mean ± SD (*n* = 3). Different superscript letters (a–d) within the same column indicate statistically significant differences among the samples, and the corresponding *p*-values are presented in the bottom row (*p* < 0.05).

**Table 3:** Comprehensive antioxidant capacity of the gluten-free extruded snacks and berry powder.

Sample	Water-Soluble Antioxidants (mg/g)	Fat-Soluble Antioxidants (mg/g)	DPPH (% Inhibition)	FRAP (mmol Fe^2+^/100 g)	*p*-Value
Corn/Rice (control)	0.031 ± 0.0002 ^c^	0.002 ± 0.0001 ^c^	18.4 ± 0.9 ^c^	0.42 ± 0.03 ^c^	<0.001
Corn/Buckwheat (control)	0.020 ± 0.0001 ^c^	0.003 ± 0.0001 ^c^	24.7 ± 1.1 ^c^	0.58 ± 0.04 ^c^	<0.001
Corn/Chickpea (control)	0.039 ± 0.0002 ^c^	0.003 ± 0.0001 ^c^	21.3 ± 1.0 ^c^	0.51 ± 0.02 ^c^	<0.001
Corn/Rice/Berry	0.71 ± 0.0085 ^b^	0.06 ± 0.0003 ^b^	61.8 ± 2.4 ^b^	1.84 ± 0.07 ^b^	<0.001
Corn/Buckwheat/Berry	0.64 ± 0.0022 ^b^	0.09 ± 0.0007 ^b^	58.9 ± 2.1 ^b^	1.71 ± 0.05 ^b^	<0.001
Corn/Chickpea/Berry	0.60 ± 0.0072 ^b^	0.08 ± 0.0009 ^b^	55.6 ± 1.8 ^b^	1.63 ± 0.06 ^b^	<0.001
Berry powder mixture	1.92 ± 0.023 ^a^	0.38 ± 0.004 ^a^	89.5 ± 3.2 ^a^	2.94 ± 0.11 ^a^	<0.001

Values are expressed as mean ± standard deviation (SD) of three replicates. Different superscript letters (a, b, c) within the same column indicate statistically significant differences across the samples according to one-way ANOVA followed by Tukey’s HSD post hoc test (*p* < 0.05).

**Table 4:** Phenolic profile of the extruded snacks and berry powder (HPLC).

Sample	Total Phenolic Content (HPLC), mg GAE/100 g	*p*-Value	Major Identified Phenolic Compounds
Corn/Rice (control)	64.8 ± 3.1 ^c^	<0.001	Trace amounts of phenolic acids
Corn/Buckwheat (control)	96.5 ± 4.4 ^c^	<0.001	Rutin, ferulic acid
Corn/Chickpea (control)	81.4 ± 3.8 ^c^	<0.001	Gallic and ferulic acids
Corn/Rice/Berry	412.6 ± 12.5 ^b^	<0.001	Anthocyanins, chlorogenic acid, quercetin
Corn/Buckwheat/Berry	395.3 ± 11.2 ^b^	<0.001	Rutin, anthocyanins, ferulic acid
Corn/Chickpea/Berry	372.8 ± 10.6 ^b^	<0.001	Gallic acid, quercetin, anthocyanins
Berry powder mixture	865.4 ± 18.7 ^a^	<0.001	Anthocyanins, quercetin, chlorogenic and gallic acids

Values are expressed as mean ± standard deviation (SD). Different superscript letters (a, b, c) within the same column indicate statistically significant differences according to one-way ANOVA and Tukey’s HSD test (*p* < 0.05).

**Table 5:** Detailed quantitative sensory evaluation of control and berry-fortified gluten-free snacks across different flour matrices (*n* = 10 assessors).

Sensory Attribute	Corn/Rice (Control)	Corn/Rice/Berry	Corn/Buckwheat (Control)	Corn/Buckwheat/Berry	Corn/Chickpea (Control)	Corn/Chickpea/Berry	*p*-Value
**Appearance (Shape and Surface)**	4.50 ± 0.10 ^b^	4.70 ± 0.10 ^a^	4.50 ± 0.10 ^b^	4.40 ± 0.10 ^b^	4.50 ± 0.10 ^b^	4.50 ± 0.10 ^b^	<0.05
**Color**	4.40 ± 0.10 ^c^	4.60 ± 0.10 ^a^	4.60 ± 0.10 ^a^	4.40 ± 0.10 ^c^	4.50 ± 0.10 ^b^	4.50 ± 0.10 ^b^	<0.001
**Texture (Crunchiness)**	4.60 ± 0.10 ^a^	4.70 ± 0.10 ^a^	4.60 ± 0.10 ^a^	4.60 ± 0.10 ^a^	4.60 ± 0.10 ^a^	4.50 ± 0.10 ^a^	0.315
**Taste and Aftertaste**	4.20 ± 0.10 ^c^	4.40 ± 0.10 ^a^	4.30 ± 0.10 ^b^	4.40 ± 0.10 ^a^	4.30 ± 0.10 ^b^	4.40 ± 0.10 ^a^	<0.01
**Aroma**	4.20 ± 0.10 ^c^	4.40 ± 0.10 ^a^	4.40 ± 0.10 ^a^	4.30 ± 0.10 ^b^	4.40 ± 0.10 ^a^	4.40 ± 0.10 ^a^	<0.01
**Total Score**	21.90 ± 0.15 ^c^	22.80 ± 0.18 ^a^	22.40 ± 0.15 ^b^	22.10 ± 0.18 ^b^	22.30 ± 0.15 ^b^	22.30 ± 0.18 ^b^	<0.001

Data are expressed as means ± standard deviations (SD). Different superscript letters (a, b, c) within the same row indicate statistically significant differences across the samples according to Tukey’s HSD post hoc test (*p* < 0.05). Control samples represent the plain extruded formulations without the addition of berry powder.

## Data Availability

The original contributions presented in the study are included in the article; further inquiries can be directed to the corresponding authors.
